# Investigating the Association between Chronic Kidney Disease and Ischaemic Stroke from a Health Examination Database

**DOI:** 10.1038/s41598-018-29161-8

**Published:** 2018-07-19

**Authors:** Chao Ou-Yang, Theresa Lalita Handaruputri, Han-Cheng Wang, Chiehfeng Chen

**Affiliations:** 1 Department of Industrial Management, National Taiwan University of Science and Technology, Taipei, Taiwan; 2 Department of Neurology, Shin Kong Wu Ho-Su Memorial Hospital, Taipei, Taiwan; 3 College of Medicine, National Taiwan University, Taipei, Taiwan; 4 College of Medicine, Taipei Medical University, Taipei, Taiwan; 5 Department of Public Health, School of Medicine, College of Medicine, Taipei Medical University, Taipei, Taiwan; 6 Cochrane Taiwan, Taipei Medical University, Taipei, Taiwan; 7 Division of Plastic Surgery, Department of Surgery, Wan Fang Hospital, Taipei Medical University, Taipei, Taiwan

## Abstract

Stroke has become one of the leading causes of death, with ischaemic stroke as the most common type of stroke occurrence compared to haemorrhagic stroke. Chronic kidney disease(CKD), another important cause of death, shares several traditional cardiovascular riskfactors with ischaemic stroke. Therefore, it is important to examine the existence of shared risk factors in the association between CKD and ischaemic stroke. This study used a health examination database from a medical centre in Taiwan. A generalized linear regression analysis was used to determine the association between CKD and ischaemic stroke. The Maentel-Haenszel test was performed to analyse the effect of possible confounding factors on the association between CKD and ischaemic stroke. A prevalence rate study showed that more subjects with CKD suffered from ischaemic stroke than subjects without CKD. Diabetes, hypertension, hypertriglyceridemia, and hypercholesterolemia were associated with increased risks of ischaemic stroke in CKD subjects. There was an inverse association of the odds ratio of ischaemic stroke between CKD and non-CKD patients, which implied that younger subjects with CKD should be made aware of ischaemic stroke prevention.

## Introduction

In the past four decades, stroke has become one of the top three leading causes of death in Taiwan^1^. Based on health databases between 2006 and 2008, the most common type of stroke in Taiwan was an ischaemic stroke (79.72%) compared to haemorrhagic stroke (20.28%)^2^.

Chronic kidney disease (CKD) is another important cause of death. In 2014, Taiwan had the highest incidence rate of end-stage renal disease in the world, with a 19.6% increase from 2001 to 2014. It was mainly attributed to the progression of CKD^3,4^.

CKD and ischaemic stroke share several traditional cardiovascular risk factors^5^. The brain and kidneys also have low vascular resistance systems in allowing continuous high-volume perfusion^6^. Although previous studies predicted an association of CKD and stroke, especially ischaemic stroke^5,7^, the nature of the association in the presence of shared risk factors should be further investigated. Risk factors of CKD and ischaemic stroke from previous studies are shown in Table 1, which also includes risk factors considered in this study. The research investigated the association of CKD and ischaemic stroke among different age groups through a health examination database.

**Table 1:** Risk factors of CKD and ischaemic stroke.

Risk Factors	Previous Studies						This study
	Sacco^13^	Grau *et al*.^14^	Li *et al*.^15^	Zhao *et al*.^16^	Bilic *et al*.^17^	Kao & Chen^18^	
Age	✓	✓	✓	✓	✓	✓	✓
Gender	✓	✓	✓	✓	✓	✓	✓
Current smoking	✓	✓	✓	✓	✓	✓	✓
Alcohol abuse^a^	✓	✓	✓	✓	✓	✓	—
Obesity	✓	✓	—	✓	—	✓	✓
Hypertension	✓	✓	✓	✓	✓	✓	✓
Diabetes mellitus	✓	✓	✓	✓	✓	✓	✓
Hypercholesterolemia^b^	✓	✓	✓	✓	✓	✓	✓
Hypertriglyceridemia^b^	✓	—	✓	✓	✓	✓	✓
Low HDL-C^b^	✓	—	✓	✓	✓	✓	✓
Race/ethnicity	✓	—	—	✓	—	—	—
Heredity	✓	—	—	✓	—	—	—
Previous stroke	—	✓	—	—	✓	—	—
Metabolic syndrome	—	—	—	✓	—	✓	—

^a^Alcohol abuse was determined when alcohol consumption exceeded 1 time/week^18^. However, all of the subject study did not consume alcohol more than 1 time/week. Therefore, alcohol abuse was not considered in this study.

^b^The previous studies actually considered hyperlipidaemia. However, because it was determined based on the occurrence of hypercholesterolemia, hypertriglyceridemia and low levels of high-density lipoprotein cholesterol (HDL-C)^19^, this study considered to separate it into those three variables.

## Methods

### Study population

This study used a health examination database from WHS Hospital in Taipei, Taiwan. Data in 2004~2011 were included. The research has the Ethical Review Approval from National Taiwan University as the number 201506EM035. The research analyzed de-identification data of a health database, so that individual patient permit was not applicable. The types of data collected in this database included a general examination, blood and urine tests, and brain magnetic resonance imaging (MRI). CKD is a general term for heterogeneous disorders affecting the structure and function of the kidneys^8^. All individuals with an estimated glomerular filtration rate (eGFR) of <60 mL/min/1.73 m^2^ for 3 months are classified as having CKD, irrespective of the presence or absence of kidney damage^9^. CKD was estimated using the four-variable Modification of Diet in Renal Disease Study Equation: eGFR(60 mL/min/1.73 m^2^) = 186.3 × serum creatinine (mg/dL)^−1.154^ × age (years)^−0.203^ × (0.742, if female)^10^. All patients received a brain MRI in the health exam. Stroke was diagnosed according to the MRI report.

### Statistical analysis

Variables in this study were divided into two types of continuous variables and categorical variables and were analyzed differently. Since the distribution of the data is unknown, nonparametric tests are used. We assumed that the distribution of the two populations (with and without ischaemic stroke) have the similar shape, Mood’s median test is a suitable method to compare continuous variables. In addition, the χ^2^ test was used to compare categorical variables for study subjects. The odds ratio is the number of those who belong to the group divided by the number of those who do not. Adjusted odds ratios were calculated using suitable statistical tests based on the amount of possible confounding factors. A value of p of <0.05 was considered statistically significant.

To choose suitable confounding factors, the Mantel-Haenszel statistical test and a generalized linear model were used. The Mantel-Haenszel statistical test was used to determine the effect of individual confounding factors with a stratification approach. Meanwhile, Meanwhile, generalized linear model was used in order to know the effect of confounders altogether in the adjustment of odds ratio. We use Logit as link function because it can support the integer input and yes/no outcome. All statistical analysis were conducted using IBM SPSS Statistics ver. 21.

## Results

### Demographics of study subjects

The study contained 13,692 health records. However, after removing duplicates and incomplete data, 12,860 data records were used in the main calculations. The distribution of CKD in various age ranges is shown in Table 2.

**Table 2:** Number of subjects in various age range.

Age Range	Subjects with CKD	Subjects without CKD
<55	58	8085
55–59	53	1961
60–64	67	1226
65–69	78	689
70–74	63	421
≥75	68	249

Further analyses of demographics, lifestyle, and biochemical factors of study subjects are described in Table 3. Among total subjects, 63.7% of patients were male. The median age of the patients was 51 years. The overall prevalence rate of ischaemic stroke was 5.8%. Subjects with ischaemic stroke were significantly older than those without ischaemic stroke (62 vs. 51 years, *p* < 0.001).

**Table 3:** Demographic, lifestyle and biochemical characteristics of the study subjects with and without ischaemic stroke.

Variable	Ischaemic Stroke	No Ischaemic Stroke	*P*
Number	748	12112	
Age	62 (15)	51 (13)	<0.001
Male (%)	70.6	63.3	<0.001
DBP (left hand)	77 (17)	73 (15)	<0.001
SBP (left hand)	134 (29)	118 (26)	<0.001
DBP(right hand)	75 (17)	72 (16)	<0.001
SBP(right hand)	132 (29)	117 (25)	<0.001
BMI	24.9 (4.3)	24.3 (4.4)	<0.001
Current smoking (%)	2.6	1.4	0.405
Fasting glucose	98 (27)	92 (14)	<0.001
Total cholesterol	204 (52.8)	198 (47.0)	0.007
HDL-C	48 (19)	50 (20)	0.001
Triglyceride	135 (19)	116 (90)	<0.001

Data are expressed as median (interquartile range).

DBP, diastolic blood pressure; SBP, systolic blood pressure; BMI, body-mass index; HDL-C, high-density lipoprotein cholesterol.

Table 3 also shows that subjects with ischaemic stroke had higher median values for blood pressure (both systolic and diastolic blood pressure, and in both the left and right hands), BMI, fasting glucose, total cholesterol, and triglycerides. Patients with ischaemic stroke had lower median values only for HDL-C. There were also more subjects with ischaemic stroke who were current smokers (2.6%) than subjects without ischaemic stroke (1.4%).

### Risk factors correlated with ischaemic stroke

Table 4 presents prevalences of risk factors in subjects with and without ischaemic stroke. Diabetes was defined as the use of medications related to diabetes treatment or a fasting blood glucose level of ≥126 mg/dL. Hypertension was defined as a systolic blood pressure (SBP) of ≥140 mmHg or a diastolic blood pressure (DBP) of ≥90 mmHg. Low HDL-C was defined as <40 mg/dL in men and <50 mg/dL in women.

**Table 4:** Prevalence of risk factors among study subjects with and without ischaemic stroke.

Variables	Ischaemic Stroke	No Ischaemic Stroke	*P*
Obesity (BMI ≥25 kg/m2)	48.1	41.7	0.001
Hypertension	48.7	22.4	<0.001
Diabetes	19.3	7.1	0.001
Total cholesterol >200 mg/dl	52.9	2.6	0.002
Triglyceride >150 mg/dl	43.0	32.4	<0.001
Low HDL-C	28.7	25.2	0.032

BMI, body-mass index; HDL-C, high-density lipoprotein cholesterol.

Compared to participants without ischaemic stroke, those with ischaemic stroke had higher rates of obesity, hypertension, diabetes, hypercholesterolemia (total cholesterol of >200 mg/dL), hypertriglyceridemia (triglycerides >150 mg/dL), and low HDL-C. The *P* values of these risk factors were less than 0.05. It indicates that statistical significant exists between the ischaemic stroke and no ischaemic stroke populations for these variables.

Table 5 presents the ORs of independent variables of ischaemic stroke. It consists of all independent variables from risk factors, lifestyle factors, and demographic variables. Based on the significance values, the difference in the likelihood of having an ischaemic stroke was statistically significant in most of the age ranges except in the range of 55–59. Other significant variables for ischaemic stroke were a male gender, obesity, hypertension, diabetes, hypertriglyceridemia, and low HDL-C. Current smoking was the only variable for which the OR was not statistically significant.

**Table 5:** Odds ratios (ORs) for ischaemic stroke.

Independent variables	OR (95% CI)
Age	
<55	0.17 (0.14–0.20)*
55–59	1.14 (0.94–1.39)
60–64	1.93 (1.57–2.38)*
65–69	3.23 (2.61–4.00)*
70–74	6.01 (4.81–7.51)*
≥75	7.04 (5.41–9.84)*
Male	1.39 (1.64–1.18)*
Current smoking	1.81 (0.43–7.59)
Obesity	1.30 (1.12–1.50)*
Hypertension	3.27 (2.82–3.8)*
Diabetes	3.13 (2.58–3.8)*
Total cholesterol >200 mg/dL	1.27 (1.09–1.47)*
Triglyceride >150 mg/dL	1.58 (1.36–1.83)*
Low HDL-C	1.20 (1.02–1.41)*

CI, confidence interval; HDL-C, high-density lipoprotein cholesterol.

*P < 0.05, statistically significant.

According to calculations of the prevalence risk and OR, hypercholesterolemia, obesity, hypertension, hypertriglyceridemia, diabetes, and low HDL-C were variables consistently achieving statistical significance. Each factor is further adjusted to analyse the possible confounding.

### Crude measurement of the association between CKD and ischaemic stroke

**Figure 1:**
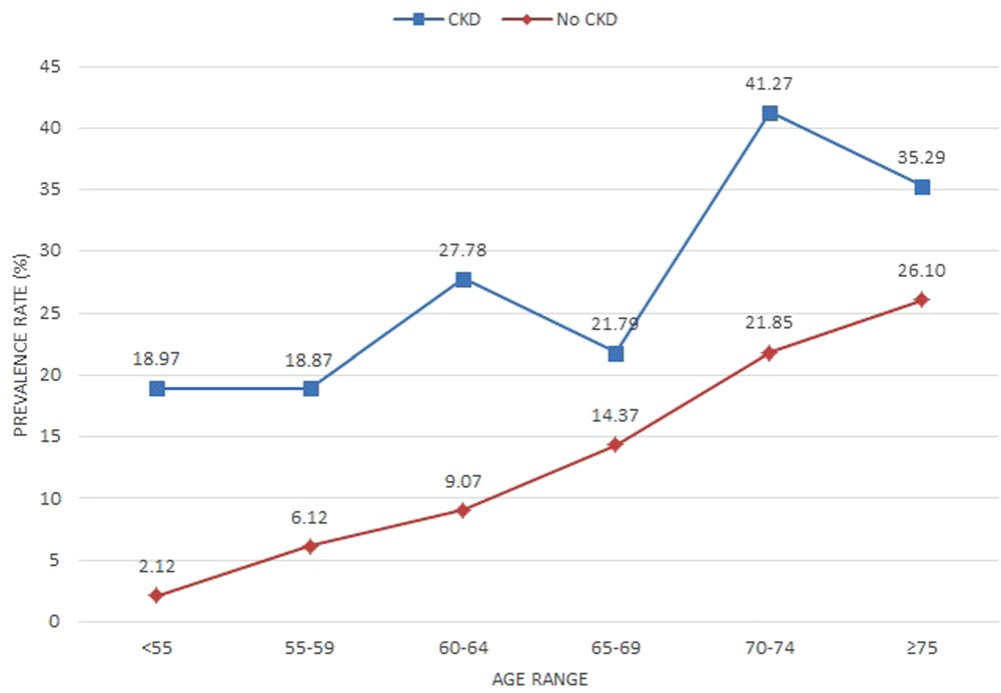
Prevalence rates of ischemic stroke of study subjects, stratified by chronic kidney disease (CKD) and age range.

A crude measurement was performed by measuring the extent of the association between CKD and ischaemic stroke without adjusting for possible confounding factors. Figure 1 describes the prevalence rate of CKD among study subjects stratified by age range. Compared to patients without CKD, CKD subjects had significantly higher prevalence rates of ischaemic stroke. Therefore, there was a greater chance for CKD patients to suffer an ischaemic stroke. A direct association with ischaemic stroke was also observed in subjects with and without CKD by age, meaning that as subjects aged, more people had a stroke.

According to the calculation of ORs of ischaemic stroke of study subjects in Table 6, there were different types of association between the likelihood of experiencing an ischaemic stroke between patients with and those without CKD. For patients with CKD, negative association between age and OR of CKD was existed, which showed high OR of ischaemic stroke for young CKD patients. Stroke made the patients more vulnerable and reduced the chance of living longer. The result implied that younger subjects with CKD should be made aware of ischaemic stroke prevention. The phenomenon is different from the non-CKD patients.

**Table 6:** Odds ratio of ischemic stroke of study subjects, stratified by CKD and age range.

Age	OR with CKD (95% CI)	OR without CKD (95% CI)
Age		
<55	10.83 (5.52–21.25)	0.09 (0.05–0.18)
55–59	3.57 (1.75–7.27)	0.28 (0.14–0.57)
60–64	3.85 (2.05–7.24)	0.26 (0.14–0.49)
65–69	1.66 (0.93–2.96)	0.60 (0.34–1.07)
70–74	2.51 (1.45–4.37)	0.40 (0.23–0.69)
≥75	1.54 (0.87–2.74)	0.65 (0.37–1.15)

### Significant confounding factors

Based on Table 7, possible confounding factors that did not reach statistical significance were obesity and low HDL-C. For this reason, only hypertension, hypercholesterolemia, diabetes, and hypertriglyceridemia were considered as significant as possible confounding factors. These factors were then analysed based on the effect of each factor in the association between ischaemic stroke and CKD. Because the occurrences of hypercholesterolemia, hypertriglyceridemia, and low HDL-C were combined in the parameter of hyperlipidaemia, the significance of their interaction was also calculated. No interactions reached statistical significance. Thus, those three variables were independent of each other. Because of that, low HDL-C was eliminated.

**Table 7:** Significance of possible confounding factors for ischaemic stroke-CKD association.

Variables	OR (95% CI)	*P*
CKD	3.60 (2.98–4.36)	<0.001
Diabetes	1.97(1.65–2.35)	<0.001
Hypercholesterolemia	1.32(1.00–1.73)	0.014
Hypertension	1.11(0.82–1.48)	<0.001
Hypertriglyceridemia	1.32(1.00–1.73)	0.046
Low HDL-C	0.93(0.77–1.11)	0.501
Obesity	0.03(0.03–0.04)	0.235
[Hypercholesterolemia = Yes]* [Hypertriglyceridemia = Yes]* [Low_HDLC = Yes]	0.74(0.45–1.22)	0.244
[Hypercholesterolemia = Yes]*[Hypertriglyceridemia = Yes]*[Low_HDLC = No]	0.87(0.62–1.23)	0.446
[Hypercholesterolemia = Yes]*[Hypertriglyceridemia = No]*[Low_HDLC = Yes]	0.65(0.37–1.14)	0.131
[Hypercholesterolemia = No]*[Hypertriglyceridemia = Yes]*[Low_HDLC = Yes]	0.86(0.56–1.33)	0.507

CI, confidence interval; HDL-C, high-density lipoprotein cholesterol.

### Magnitude of confounding

According to LaMorte & Sullivan^11^, a simple and direct way to determine whether a given risk factor causes confounding is to compare the estimated measure of association before and after adjusting for confounding. This is done by computing the measurement of the association both before and after adjusting for a potential confounding factor. If the difference between the two measures of association is ≥10%, then confounding is present.

Table 8 describes comparisons of crude ORs and adjusted ORs of ischaemic stroke in terms of diabetes, hypertension, hypertriglyceridemia, and hypercholesterolemia. It shows that all of the possible confounding factors had an average value of magnitude of confounding that surpassed the threshold value of 10%. Therefore, diabetes, hypertension, hypertriglyceridemia, and hypercholesterolemia were confounding factors in the association between CKD and ischaemic stroke.

**Table 8:** Magnitude of confounding by possible confounding factors for CKD patients.

Age	Crude (95%CI)	Diabetes		Hypertension		Hypertriglyceridemia		Hypercholesterolemia	
		Adjusted (95%CI)	Magnitude of confounding	Adjusted (95%CI)	Magnitude of confounding	Adjusted (95%CI)	Magnitude of confounding	Adjusted (95%CI)	Magnitude of confounding
<55	10.83 (5.52–21.25)	7.11 (4.06–12.45)	34.36%	5.86 (3.41–10.09)	45.90%	8.12 (4.72–13.97)	25.03%	8.87 (5.12–15.35)	18.11%
55–59	3.57 (1.75–7.27)	2.77 (1.57–4.87)	22.36%	3.25 (1.85–5.69)	8.91%	2.82 (1.57–5.08)	20.96%	3.06 (1.7–5.49)	14.23%
60–64	3.85 (2.05–7.24)	2.88 (1.79–4.62)	25.27%	2.75 (1.73–4.37)	28.65%	2.9 (1.83–4.61)	24.75%	3.1 (1.94–4.95)	19.56%
65–69	1.66 (0.93–2.96)	1.5 (0.95–2.36)	9.69%	1.48 (0.94–2.33)	10.89%	1.42 (0.89–2.28)	14.50%	1.52 (0.96–2.4)	8.48%
70–74	2.51 (1.45–4.37)	1.74 (1.18–2.05)	30.76%	1.91 (1.36–2.69)	23.99%	1.29 (1.23–2.67)	48.67%	1.89 (1.34–2.67)	24.79%
≥75	1.54 (0.87–2.74)	1.29 (0.32–1.95)	16.45%	1.3 (0.87–1.96)	15.81%	1.29 (0.86–1.94)	16.45%	1.3 (0.87–1.95)	15.81%
**Average**			**23.15%**		**22.36%**		**25.06%**		**16.83%**

After adjusting for each confounding factor, OR values dropped in CKD subjects, meaning that the presence of each confounding factor increased the likelihood of having an ischaemic stroke in CKD subjects. An negative association between ischaemic stroke and CKD occurred both in the crude and adjusted measurements.

### Comparison of crude and adjusted ORs of ischaemic stroke

Table 9 shows crude and adjusted ORs of ischaemic stroke among CKD subjects stratified by age range. Adjustments were made to eliminate the presence of all confounding factors from the association.

**Table 9:** ORs of ischaemic stroke, stratified by age range among CKD patients.

	Crude	Adjusted
Age Range	OR (95% CI)	OR (95% CI)
<55	10.83 (5.52–21.25)*	4.53 (2.61–7.84)*
55–59	3.57 (1.75–7.27)	3.09 (1.46–6.54)*
60–64	3.85 (2.05–7.24)*	3.52 (1.84–6.71)*
65–69	1.66 (0.93–2.96)	1.5 (0.83–2.69)
70–74	2.51 (1.45–4.37)*	2.34 (1.33–4.14)*
≥75	1.54 (0.87–2.74)	1.31 (0.89–1.93)

*P < 0.05, statistically significant.

Adjustment for all confounding factors showed that OR values dropped in CKD subjects. This result showed that the presence of all confounding factors increased the likelihood of having an ischaemic stroke in CKD subjects. An negative association between ischaemic stroke and CKD occurred in both the crude and adjusted measurements.

## Discussion

There were two main results for prevalence rates (both for the crude and adjusted measurements). The risk of ischaemic stroke in CKD patients was greater than that in non-CKD patients. A direct negative association also occurred between stroke and CKD, meaning that as subjects aged, more people had a stroke.

For the OR, there were two main results in both the crude and adjusted measurements. The first result was that patients with CKD were more likely to have an ischaemic stroke. Next, there was an inverse association of the OR of ischaemic stroke between CKD and non-CKD subjects, which implied that younger subjects with CKD should be made aware of ischaemic stroke prevention.

This study compared the characteristics of study subjects with and without ischaemic stroke. Then, prevalence of risk factors among subjects with and without ischaemic stroke were showed. Exposure to and outcomes of ischaemic stroke were then calculated using a multivariate analysis of the ORs. Those calculations were utilized to determine the prevalence rate and OR of ischaemic stroke of study subjects with CKD. Possible confounding factors were also identified by comparing the crude and adjusted ORs to find the type of association between ischaemic stroke and CKD. Diabetes, hypertension, hypercholesterolemia, and hypertriglyceridemia were confounding factors for the association between ischaemic stroke and CKD. These variables of diseases also could be considered as risk markers rather than exposure factors as our study was follow up but a cross-sectional study. The presence of confounding factors increased the likelihood of ischaemic stroke in CKD subjects.

Thus, this study recommended that in general, attention should be given to older patients since more of them suffer from an ischaemic stroke than younger patients. However, for young CKD patients, the risk of ischaemic stroke should not be ignored. The presence of diabetes, hypertension, hypertriglyceridemia, and hypercholesterolemia in patients should also be taken into account, since they increase the likelihood of having an ischaemic stroke.

The mechanism behind the association of CKD and ischaemic stroke may be carotid stenosis. Yu *et al*.^12^ found that CKD is an independent predictor of carotid plaque, stenosis, and occlusions in patients with acute stroke. They found that the eGFR was negatively correlated with the degree of carotid stenosis (p < 0.05). The finding sheds light on a possible stroke assessment of checking the intima-media thickness (IMT) of the carotid artery, especially in CKD patients.

This study has some limitations. First, selection of independent variables was based on limited knowledge, by examining the previous literature. Second, this study used a cross-sectional design which did not allow us to identify causal relationships among ischaemic stroke, CKD, and confounding factors. Despite these limitations, this study provides important information about ischaemic stroke in CKD patients.

## Conclusions

This study recommends that in general, attention should be given to older patients, since more of them suffer from ischaemic stroke than younger patients. The odds ratio of ischaemic stroke increased when the non-CKE subjects in the aging process. Thus, there was a decreasing of the odds ratio of ischaemic stroke when CKD patients become older, which implied that younger CKD patients should be made aware of ischaemic stroke prevention. The presence of diabetes, hypertension, hypertriglyceridemia, and hypercholesterolemia in patients should also be taken into account, since they increase the likelihood of having an ischaemic stroke.
